# Sensitive and Selective NH_3_ Monitoring at Room Temperature Using ZnO Ceramic Nanofibers Decorated with Poly(styrene sulfonate)

**DOI:** 10.3390/s18041058

**Published:** 2018-04-01

**Authors:** Rafaela S. Andre, Dongwook Kwak, Qiuchen Dong, Wei Zhong, Daniel S. Correa, Luiz H. C. Mattoso, Yu Lei

**Affiliations:** 1 Department of Chemical and Biomolecular Engineering, University of Connecticut, 191 Auditorium Road, Storrs, CT 06269, USA; lela_rsa@hotmail.com; 2 Nanotechnology National Laboratory for Agriculture (LNNA), Embrapa Instrumentação, São Carlos 13560-970, SP, Brazil; luiz.mattoso@embrapa.br; 3 PPGQ, Department of Chemistry, Center for Exact Sciences and Technology, Federal University of São Carlos (UFSCar), São Carlos 13565-905, SP, Brazil; 4 Department of Materials Science and Engineering, University of Connecticut, 97 North Eagleville Road, Storrs, CT 06269, USA; dongwook.kwak@uconn.edu; 5 Department of Biomedical Engineering, University of Connecticut, 260 Glenbrook Road, Storrs, CT 06269, USA; qiuchen.dong@uconn.edu; 6 Department of Chemistry, University of Connecticut, 55 North Eagleville Road, Storrs, CT 06269, USA; wei.zhong@uconn.edu

**Keywords:** ZnO NFs, PSS, NH_3_ sensor, room temperature sensing, good sensitivity

## Abstract

Ammonia (NH_3_) gas is a prominent air pollutant that is frequently found in industrial and livestock production environments. Due to the importance in controlling pollution and protecting public health, the development of new platforms for sensing NH_3_ at room temperature has attracted great attention. In this study, a sensitive NH_3_ gas device with enhanced selectivity is developed based on zinc oxide nanofibers (ZnO NFs) decorated with poly(styrene sulfonate) (PSS) and operated at room temperature. ZnO NFs were prepared by electrospinning followed by calcination at 500 °C for 3 h. The electrospun ZnO NFs are characterized to evaluate the properties of the as-prepared sensing materials. The loading of PSS to prepare ZnO NFs/PSS composite is also optimized based on the best sensing performance. Under the optimal composition, ZnO NFs/PSS displays rapid, reversible, and sensitive response upon NH_3_ exposure at room temperature. The device shows a dynamic linear range up to 100 ppm and a limit of detection of 3.22 ppm and enhanced selectivity toward NH_3_ in synthetic air, against NO_2_ and CO, compared to pure ZnO NFs. Additionally, a sensing mechanism is proposed to illustrate the sensing performance using ZnO NFs/PSS composite. Therefore, this study provides a simple methodology to design a sensitive platform for NH_3_ monitoring at room temperature.

## 1. Introduction

Ammonia (NH_3_) is of great commercial interest due to its unique properties for applications as nitrogen source for soil fertilizers, neutralizing agent in the oil industry, gas refrigerant in industrial systems and many uses in pharmaceutical industries [1,2]. However, it is also well documented that ammonia is a toxic gas for human and animals in concentrations above 25 ppm [3,4]. Long-term exposure to NH_3_ at an unsafe level could cause pulmonary and lung diseases to human and animals besides other health issues [5,6]. Consequently, it is of paramount importance to monitor and control the ammonia levels in closed environments at ambient temperatures, such as in poultry farms [7,8,9,10]. The development of devices capable of operating at ambient temperature is of great interest due to features such as the reduced power consumption, simple operational setup, and longer lifetime of device. Besides, devices operating at ambient temperature present a high potential of portability and integration for smart systems [11,12,13]. Another important remark on the application of such devices, besides poultry farms safety, is the possibility of noninvasive disease diagnoses through gas detection using human breath at ambient temperatures. Therefore, there is a huge demand in the development of novel and reliable gas sensors operating at room temperature for various applications [14,15,16,17,18].

Due to the toxicity of NH_3_ even at low concentration as aforementioned, the development of gas sensors capable to sensitively and selectively detect low concentrations of NH_3_ at room temperature [19,20,21,22,23,24] can play an important role in poultry farms, to guarantee safety and nontoxic environment, as well in health care by noninvasive diseases diagnoses. In the past decade, ceramic materials as inorganic oxide semiconductors have been extensively explored as gas sensing materials because of the changes in their electrical characteristic clearly observed upon the exposure to reducing/oxidizing gases [25,26,27,28]. One of the best known semiconductors is n-type ZnO, a material chemically stable with high electron mobility and conductivity, which can be easily obtained through inexpensive methods [29,30,31,32,33,34,35]. However, most of ZnO based gas sensors reported in the literature are obtained as thin film and/or operated at elevated temperatures. Still, thin-film based metal oxides as sensing materials saturate quickly and also suffer from the strong diffusion resistance, which typically result in long response/recovery time and low sensitivity [36]. Furthermore, the operation at an elevated temperature requires high-energy consumption. As a good alternative architecture, the sensing materials in nanofibers configuration is more attractive in gas sensor application when compared with thin films because porous nanofiber materials possess both high surface area and good gas accessibility, thus resulting in rapid response and good sensitivity [37,38,39,40]. Moreover, the association of ZnO with different materials for *p*-*n* junction fabrication has been reported in the literature in order to overcome some drawbacks, which include: limitation of operation at room temperature (quick saturation), high response time and low stability [41,42,43,44,45]. For example, Wang et al. [22] reported a high performance NH_3_ sensor based on NiO/ZnO heterostructure. The authors presented the ZnO *n*-type association with the NiO *p*-type as a formation of *p-n* junction. Once ZnO has electron as the majority of the charge carriers it promoted the charge transfer and a reduced potential barrier speeding up the gas adsorption on the material surface. As a consequence, the sensor presented a lower dependence on the temperature and humidity when compared with pure NiO. Another great combination for *p-n* junction is the use of conjugated polymers with inorganic semiconductors. Pang et al. [46] described the fabrication of a cellulose/TiO_2_/polyaniline (cellulose/TiO_2_/PANI) composite with much higher response values to NH_3_ sensing than cellulose/PANI composite. The presence of TiO_2_ improved the negative charge carrier concentration, thus resulting in improved charge transfer and the amplified resistance variation.

In this paper, we report a facile method to prepare a NH_3_ gas sensing composite, composed of ZnO nanofibers (ZnO NFs) decorated with poly(styrene sulfonate) (PSS). ZnO NFs were obtained by electrospinning method followed by calcination treatment in order to generate inorganic phase crystallization and remove polymer matrix. Although different polymers, such as poly(3-hexylthiophene) and PANI, have been employed to enhance the NH_3_ sensing performance, PSS was chosen for coating the ZnO NFs in this study because it is a cost-effective polyelectrolyte capable of promoting an improved adhesion of the sensitive layer onto the substrate [47] and the charge transfer from the sensitive layer to the interdigitated electrode, and also possesses numerous sulfonated groups (SO_3_^−^), which favors the ammonia interaction with the sensitive layer. The PSS decoration onto ZnO NFs was carried out by a simple mixing/casting method. Various advanced characterization techniques were carried out to analyze the as-prepared ZnO NFs and the ceramic nanometric fibers morphology. The ZnO NFs coated with an optimized amount of PSS were employed to bridge an interdigitated gold electrode and then investigated for low concentrations of ammonia monitoring at room temperature.

## 2. Materials and Methods

### 2.1. Reagents

Poly(vinyl pyrrolidone) (PVP, Mw = 1,300,000.00 g mol^−1^), zinc nitrate hexahydrate (Zn(NO_3_)_2_·6H_2_O), dimethylformamide (DMF) and poly(styrene sulfonate) (PSS, Mw= 200,000 g·mol^−1^) solution 30 wt % in H_2_O were all purchased from Sigma-Aldrich (St. Louis, MO, USA). The gas sensing experiments were performed using synthetic dry air, ammonia gas mixture (NH_3_, 200 ppm in air), nitrogen dioxide (NO_2_, 1000 ppm in N_2_) and carbon monoxide (CO, 5% in Argon) obtained from Airgas (Radnor, PA, USA).

### 2.2. ZnO Nanofibers Fabrication

ZnO NFs were fabricated by electrospinning followed by calcination using a modified procedure reported in our previous works [48,49,50]. Briefly, Zn(NO_3_)_2_·6H_2_O and PVP (50:50 wt %) were dissolved in 3 mL DMF. The precursor solution was stirred for 6 h and then transferred to a plastic syringe with a 19-gauge needle for the electrospinning process. Electrospinning parameters were set as 20 kV of the applied voltage, a flow rate of 0.3 mL h^−1^ and 15 cm of the collecting distance. The collected nanofibers were subjected to a thermal treatment in a muffle furnace at 500 °C for 3 h for the PVP matrix removal and the formation of ZnO NFs.

### 2.3. Characterization

The structural crystalline phase composition of the as-prepared ZnO nanofibers was characterized by X-ray diffraction (XRD) acquired with an Oxford diffraction XcaliburTM PX Ultra with ONYX detector. The morphology and the size of the electrospun ZnO nanofibers after calcination were observed through scanning electrons microscopy (SEM) using a JEOL 6335F field-emission scanning electron microscope. The nanofiber diameters and their size distribution were estimated using an image analysis software (Image J, National Institutes of Health, Bethesda, MD, USA). For further study of the chemical composition and surface characteristics of the obtained ceramic nanofibers, X-ray photoelectron spectroscopy (XPS) was carried out with a PHI multiprobe using Mg K (1253.6 eV) as the exciting source.

### 2.4. Sensor Fabrication and Gas Testing System

To prepare the ZnO NFs functionalized with PSS, ZnO NFs suspension was mixed with PSS aqueous solutions. The final concentration of ZnO NFs was 5 mg mL^−1^ with the different ratios (by weight percentage) of ZnONFs:PSS at 94:6, 75:25 and 50:50, respectively. A 5 mg mL^−1^ of ZnO NFs aqueous suspension without PSS was also used to prepare the control sensor for a comparative purpose. The suspensions were sonicated for 30 min and then 2 μL of each ZnO NFs/PSS suspension was drop-cast on a gold interdigitated electrode (IDE). The IDEs were fabricated on silicon wafer by photolithography using a layer of chromium followed by gold layer deposition. The fabricated IDEs possessed 40 fingers, having both finger width and gap between fingers of 3 µm. After solvent evaporation, the NH_3_ sensors were ready for use.

**Figure 1:**
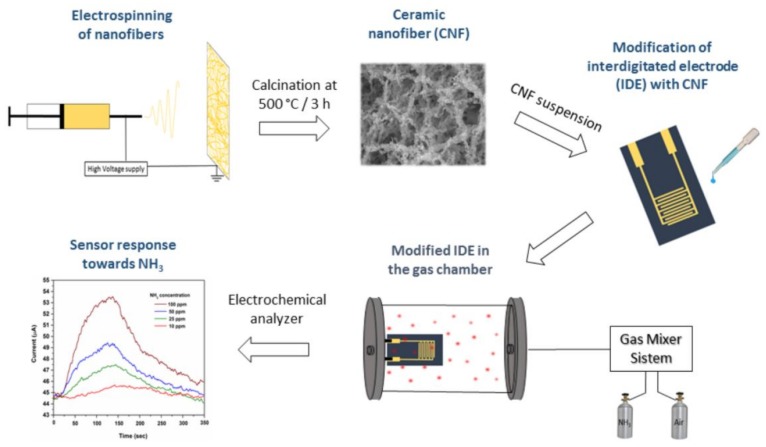
Schematic representation of the ceramic nanofibers (CNF) fabrication deposited onto gold interdigitated electrode (IDE) and the system for NH_3_ monitoring tests.

The sensor devices prepared with different ratios of ZnO NFs and PSS (94:6, 75:25 and 50:50) are referred to as ZnO94/PSS6, ZnO75/PSS25 and ZnO50/PSS50 in the subsequent discussion. In a typical NH_3_ sensing experiment, the sensor device was placed in a tube chamber and connected to a CHI 660D electrochemical analyzer (CH Instruments Inc., Austin, TX, USA). The current output was optimized and fixed at 5 V DC bias for continuously measurement. A schematic representation of the ceramic nanofiber fabrication deposited onto gold interdigitated electrode (IDE) and the system for NH_3_ monitoring tests is displayed in Figure 1.

### 2.5. NH_3_ Sensing at Room Temperature

The performance of ZnO NFs/PSS based gas sensor at room temperature (25 °C) was evaluated by measuring the resistance change upon exposure to different concentrations of NH_3_ in a dynamic gas flow system and synthetic air was used as the carrier gas. The gas flow rate was set as 3 L min^−1^ for the carrying synthetic air and gas mixtures (NH_3_/synthetic air). Gas flow and target gas concentration in ppm were regulated automatically using a computer-controlled gas mixing system (S-4000, Environics Inc., Tolland, CT, USA). Prior to the measurement, the carrying gas was purged for 40 min in order to obtain a stable baseline. For all the NH_3_ sensing experiment, the sensor was exposed to NH_3_ for 4 min followed by synthetic air for 6 min to recover the sensor, and then the procedure was repeated for multiple cycles. The current in the sensor was continuously measured during the gas flow cycles. The electric resistance of the sensor was calculated by applying Ohm’s Law (*R* = *V*/*I*) and the resistance variation observed could be directly related to the ammonia concentration and normalized as Δ*R*/*R*_0_% = [(*R*_0_ − *R_g_*)/*R*_0_] × 100%, where *R*_0_ is the electrical resistance of the sensor in synthetic air and *R_g_* is the measured resistance in NH_3_.

## 3. Results and Discussion

### 3.1. Structural and Morphological Characterization of ZnO NFs

**Figure 2:**
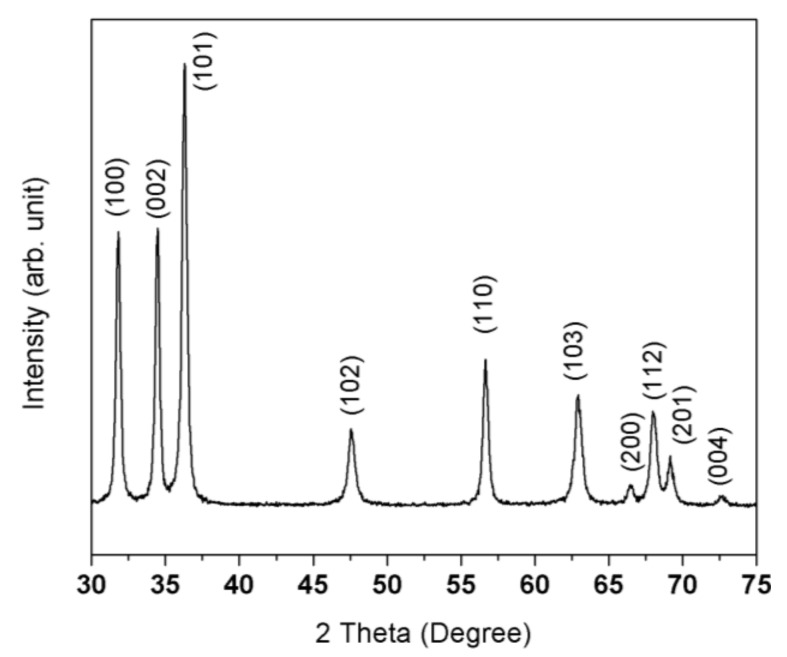
X-ray diffraction pattern of the as-prepared ZnO nanofibers.

Powder X-ray diffractometry (XRD) was employed to confirm the crystal structure of the as-prepared ZnO NFs. Figure 2 shows the XRD pattern of ZnO nanofibers after calcination. One can see that ZnO NFs are crystalline and all diffraction peaks can be assigned to hexagonal wurtzite ZnO phase according to the JCPDS Card No. 36-1451. No diffraction peaks related to secondary phases were found, indicating that all organic and polymeric materials were decomposed during the thermal treatment and thus no impurities or secondary phases are present in the nanofibers.

**Figure 3:**
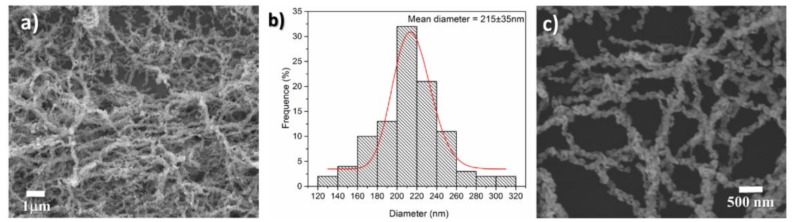
(**a**) Scanning electron microscopy picture of ZnO nanofibers; (**b**) a Histogram of diameter size distribution; (**c**) ZnO nanofibers at higher magnification.

The nanofibers morphology of the as-prepared ZnO was confirmed using SEM. As shown in Figure 3a, the calcined ZnO product displayed a nanofibrous structure with a mean diameter of 215 nm (Figure 3b). More interestingly, the as-prepared ZnO NFs consisted of numerous interconnected spherical nanoparticles with a mean diameter of 75 nm, as shown in Figure 3c under a higher magnification. Such unique nanoparticle-necklaced nanofiber structure endows the as-prepared ZnO possessing very large surface area and high porosity, which is favorable for subsequent gas sensing.

**Figure 4:**
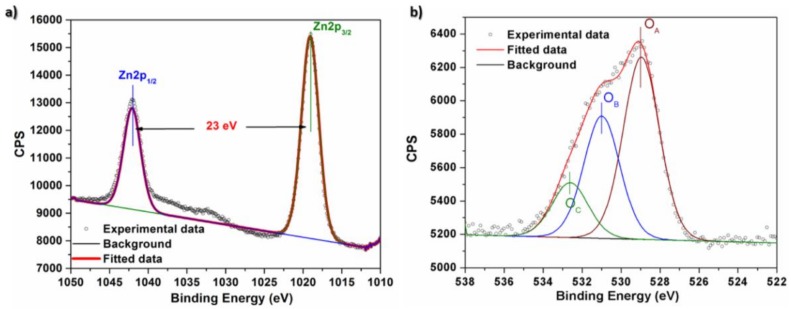
The high-resolution X-ray photoelectron spectroscopy (XPS) spectra of ZnO NFs (**a**) in the Zn 2p region and (**b**) the O 1s region.

The composition and chemical bond configuration of the as-prepared ZnO NFs were further studied through X-ray photoelectron spectroscopy (XPS). The XPS data was corrected with respect to the standard peak of C 1s at 284.6 eV. The main peaks in XPS survey spectrum of ZnO NFs (data not shown) can be assigned to Zn and O bands and no other peak was observed, indicating high chemical purity of the as-prepared ZnO NFs. Figure 4a presents high resolution spectrum of Zn 2p region with two peaks located at 1042.08 eV and 1019.08 eV (corresponding to Zn 2p_1/2_ and Zn 2p_3/2_, respectively), indicating that all Zn ions in the sample are in Zn^2+^ oxidation state and correspond to a 2p binding energy of Zn(II) ions [51,52]. The 23 eV of distance between these two peaks is in good agreement with the splitting energy reported in the literature for ZnO [51,52]. The high resolution O 1s spectrum also shows its characteristics binding energy. Three distinct oxygen species for O 1s could be well distinguished in Figure 4b. The main peak at 528.95 eV corresponds to the ions in the ZnO crystal lattice (O_A_) while the peak at 530.98 eV can be related to oxygen vacancies in the crystal lattices (O_B_). The peak at 532.61 eV can be ascribed to oxygen adsorbed on the ZnO surface mainly in the form of hydroxide (–OH), but also as –CO_3_, H_2_O, and O_2_^−^ species [53,54].

### 3.2. Gas Sensing Studies

**Figure 5:**
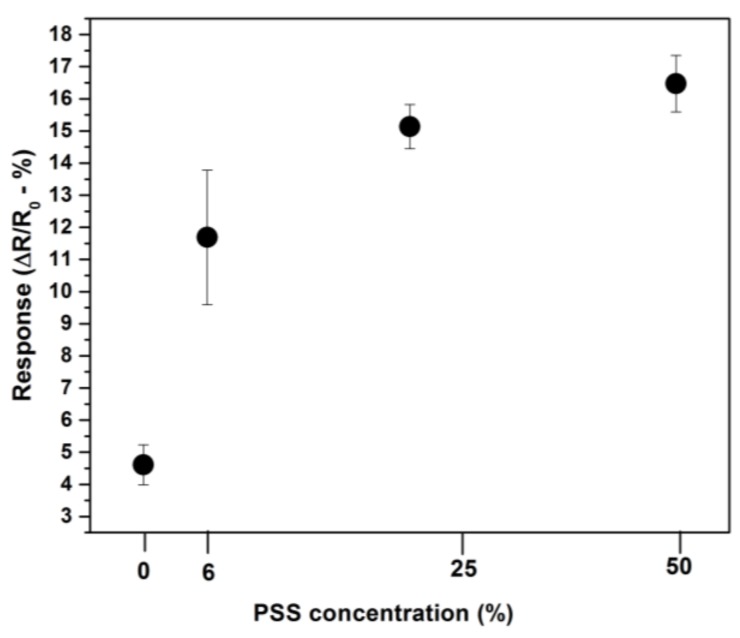
The normalized sensing response of different devices exposed to 100 ppm of NH_3_ as a function of poly(styrene sulfonate) (PSS) concentration in: pure ZnO NFs (0% of PSS), ZnO94/PSS6 (6% of PSS), ZnO75/PSS25 (25% of PSS) and ZnO50/PSS50 (50% of PSS) devices.

The as-prepared devices (ZnO94/PSS6, ZnO75/PSS25 and ZnO50/PSS50) were tested for ammonia (NH_3_) sensitivity at room temperature in order to optimize the loading of PSS. A device prepared with pure ZnO nanofibers without PSS (denoted as PZnO) was also tested for NH_3_ sensitivity for comparison, in order to illustrate the role of PSS. It is worth noting that PSS is non-conducting, thus itself cannot be used to fabricate resistor-type gas sensor. Figure 5 shows the response of different devices with distinct ZnO/PSS ratio upon the exposure to 100 ppm of NH_3_ at room temperature. One can see that PZnO-based device only possesses very low response to NH_3_. After the decoration of ZnO NFs with PSS, the sensor response to NH_3_ increases significantly. Overall, the sensor response to NH_3_ increases proportionally to the PSS concentration and then gradually levels off. When the percentage of PSS in the composite is over 50%, the sensor displays poor conductivity as well as high background noise (data not shown). To balance the sensing performance and also the conductivity/noise of the device, the ZnO50/PSS50 was then chosen for subsequent NH_3_ sensing experiments at room temperature.

**Figure 6:**
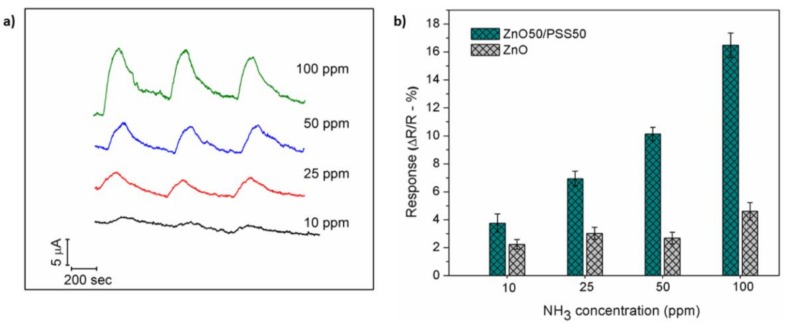
(**a**) Response-recovery profile of ZnO50/PSS50 device as a function of time for NH_3_ concentration varying from 10 to 100 ppm, and (**b**) sensing response as a function of NH_3_ concentration for ZnO50/PSS50 and PZnO devices, respectively.

Figure 6a presents the response-recovery profile of ZnO50/PSS50 to various NH_3_ gas concentrations (10, 25, 50 and 100 ppm, using air as the carrying gas) as a function of time. One can see that the supply of NH_3_ gas results in a rapid current increase, in good agreement with a typical *n*-type semiconductor sensing mechanism. After purging with the carrying air, the signal of the sensing device gradually returns to the baseline level, indicating good recovery capability of the sensor. Figure 6b compares the sensing response of PZnO and ZnO50/PSS50 for different concentrations of NH_3_. As can be observed, the ZnO50/PSS50 possesses significantly enhanced sensing performance than PZnO across all tested NH_3_ concentrations. The enhanced performance can be attributed to the presence of PSS on ZnO NFs. Firstly, PSS possesses –SO_3_^−^ groups, thus the strong interaction between –SO_3_^−^ groups and basic NH_3_ can absorb more NH_3_ to the close contact with ZnO NFs and change the charge state of PSS, enhancing the sensing performance. Secondly, the PSS is a well-known polyelectrolyte with ionic groups that enable/increase the charge mobility. Therefore, the presence of PSS could improve the charge mobility between the ZnO ceramic nanofibers and the target [55], thus enhancing the sensing performance. In addition, the small error bar calculated from three successive cycles of sensing tests indicates the good stability/reproducibility of the sensing composite, as well as its good reversibility for the NH_3_ adsorption/desorption process.

As shown in Figure 6b, the ZnO50/PSS50 sensor response for different NH_3_ concentrations showed a dynamic linear range up to 100 ppm (*R*^2^ = 0.9807). The theoretical limit of detection (LOD) was calculated as 3.22 ppm based on signal to noise ratio of 3 (S/N = 3). It is worth noting that the response-recovery profile for 10 ppm of NH_3_ seems to be low in Figure 6a. This is because of the co-display of all response-recovery profiles at different concentrations in one plot with the same scale. For better quantitative representation, Figure 6b confirms the good sensing performance even for 10 ppm of NH_3_ with a prominent response of 4.61 ± 0.62%, indicating that the developed sensor is well-positioned for the detection of NH_3_ lower than 25 ppm, the recommended limit for long period of exposure for human and animals [3,4]. Such sensing response at room temperature also indicates that the as-developed NH_3_ sensing platform possesses an outstanding performance compared with other room-temperature NH_3_ gas sensors reported in the literature, such as sensors based on pure ceramic nanoparticles [32,56]. To the best of our knowledge, ZnO NFs/PSS composition for ammonia sensing at room temperature has not been reported in the literature before. This study provides insightful information for the development of composite gas sensors based on ceramic nanofibers/polymer suitable for operation at ambient temperature with high performance. Table 1 brings a comparison of the performance of the developed sensor with other NH_3_ sensing devices reported in the literature for hybrid materials operating at room temperature in terms of response magnitude and response/recovery time.

**Figure 7:**
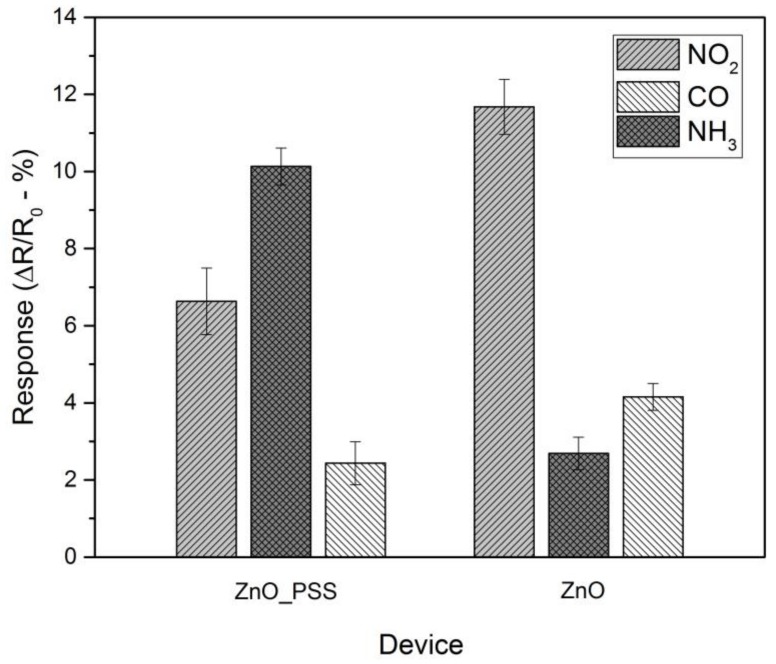
Selectivity study of ZnO50/PSS50 and PZnO devices upon exposure to 50 ppm of NO_2_, NH_3_ and CO, respectively.

The selectivity of the ZnO50/PSS50 and PZnO were also investigated and the corresponding results are presented in Figure 7. The sensor devices were tested for 50 ppm of NO_2_ and CO, which are potential interferents in ammonia sensing in indoor environment at poultry farms [7,60]. According to the absolute value of the normalized sensor response shown in Figure 7, the PZnO device showed poor selectivity against NO_2_ and CO. The decoration of ZnO NFs with PSS could significantly improve the sensor response to NH_3_ compared to NO_2_ and CO, though the interference from NO_2_ and CO cannot be fully eliminated. Such enhanced selectivity of the developed sensor can be attributed to the improved response to NH_3_ and the reduced response to NO_2_ and CO because of the presence of PSS (Figure 7).

The long-term stability is another important analytical characteristic to evaluate the sensor performance, therefore, long-term stability of the ZnO50/PSS50 sensor device was investigated over 40 days. The results showed that the ZnO NFs coated with PSS has reasonably good stability in ambient atmosphere and the sensor still possesses 70% of the original response after 40 days. 

### 3.3. NH_3_ Sensing Mechanism

The selectivity and sensitivity of semiconductor-based gas sensor is greatly related with the oxide grain size, assuming the grain boundaries acting as scattering centers for the electrons [36,61] as well as the materials surface functionality and temperature. Recognized as an effective material for gas detection at temperatures above 150 °C [62,63,64,65,66], ZnO semiconductor are able to interact with the target gas through several events since the thermic energy coming from heating could reduce the potential barrier, thus enabling the electron flow and so the gas detection [67]. Therefore, the excellent sensitivity and good selectivity of ZnO50/PSS50 at room temperature can be attributed to the high surface area and grain boundary interconnections observed for ZnO nanofibers and presented in Figure 3a. The high surface area and grain boundary interconnections indicate more abundant scattering centers. In conjunction with the charge mobility improvement through the sensitive layer/substrate promoted by the polyelectrolyte PSS, ZnO NFs/PSS offers us a sensing material with unique properties for gas sensors application. As confirmed by XPS characterization, the ZnO nanofiber surface presents O_2_^−^ adsorbed species. After coating with PSS, a heterojunction is formed at the ZnO-PSS interface. Therefore, the enhanced sensing performance can be rationalized by the effects of the PSS presence in multiple steps: the electron carrier in ZnO NFs is depleted by the proton-donating PSS at the interface, which, in turn, induces a smaller carrier base and thus an amplified sensing response [68]. In addition, the NH_3_ adsorption can be proposed by a mechanism of interaction with the SO_3_^−^ groups from PSS, which increases the charge carrier concentration decreasing the resistivity [55,69,70]. Moreover, an electrostatic potential gradient, or local electrostatic field, is established at the depletion layer, which favors the charge transport [68]. This likely accelerates the adsorption of the polar gases (e.g., NH_3_). As both adsorption of analyte species and electron transition between the analyte and sensing materials have been enhanced, the formation of PSS-ZnO heterojunction is favorable for the electronic gas sensing. Consequently, the obtained ZnO50/PSS50 based sensor could detect low concentration NH_3_ sensitively at room temperature.

## 4. Conclusions

A simple and sensitive resistor-type NH_3_ gas sensor based on a novel ZnO NFs/PSS composite was successfully developed and operated at room temperature. ZnO NFs were prepared by electrospinning followed by calcination. PSS polyelectrolyte was employed to decorate ZnO NFs to improve its adhesion to the IDE electrodes and also serve as a dopant to form heterojunction at the interface between ZnO NFs and PSS, thus resulting in an enhanced sensing performance. Upon exposure to NH_3_ gas, both rapid response and fast recovery can be achieved with an dynamic range up to 100 ppm and LOD of 3.22 ppm (S/N = 3). The presence of PSS also significantly improves the selectivity against NO_2_ and CO, compared to the device fabricated only using ZnO NFs. This work demonstrates that PSS is suitable for the tuning of the electronic structures of ZnO NFs, thus offering a facile way to develop room-temperature gas sensor with high performance.

## Figures and Tables

**Table 1:** NH_3_ sensing properties of reported sensors based on hybrid materials.

Material	Response (Δ*R*/*R_g_*)	Response Time (s)	Recovery Time (s)	NH_3_ Concentration	Reference
ZnO/PSS nanofibers	17%	51	160	100 ppm	This work
Polyaniline (PANI)/SnO_2_	-	33	-	100 ppm	[57]
ZnO	10%	49	19	100 ppm	[58]
Cellulose/TiO_2_/PANI nanofibers	3.5	83	130	100 ppm	[46]
rGO/AgNWs *	15%	60	150	100 ppm	[59]
rGO/Co_3_O_4_ nanofibers	53.6%	4	300	50 ppm	[27]

* rGO/AgNWs: reduced graphene oxide with silver nanowires.

## References

[1] Timmer B., Olthuis W., Berg A. (2005). Van Den Ammonia sensors and their applications—A review. Sens. Actuators B Chem..

[2] Wang Q., Dong X., Pang Z., Du Y., Xia X., Wei Q., Huang F. (2012). Ammonia Sensing Behaviors of TiO_2_-PANI/PA6 Composite Nanofibers. Sensors.

[3] Griffiths R.F., Megson L.C. (1984). The effect of uncertainties in human toxic response on hazard range estimation for ammonia and chlorine. Atmos. Environ..

[4] Corkery G., Ward S., CKenny C., Hemmingway P. (2013). Title Monitoring environmental parameters in poultry production facilities Author(s). Computer Aided Process Engineering, CAPE Forum 2013.

[5] Mani G.K., Rayappan J.B.B. (2015). A highly selective and wide range ammonia sensor—Nanostructured ZnO:Co thin film. Mater. Sci. Eng. B.

[6] Ghosh R., Nayak A.K., Santra S., Pradhan D., Guha P.K. (2015). Enhanced ammonia sensing at room temperature with reduced graphene oxide/tin oxide hybrid films. RSC Adv..

[7] Bittman S., Jones K., Vingarzan R., Hunt D.E., Sheppard S.C., Tait J., So R., Zhao J. (2015). Weekly agricultural emissions and ambient concentrations of ammonia: Validation of an emission inventory. Atmos. Environ..

[8] Ji B., Gates R.S., Zheng W., Grift T.E., Green A.R., Koelkebeck K.W. (2015). Design and Performance Evaluation of Upgraded Portable Monitoring Units for Barn Air Quality. ASABE Annu. Int. Meet..

[9] Zhao Y., Shepherd T.A., Li H., Xin H. (2015). Environmental assessment of three egg production systems—Part I: Monitoring system and indoor air quality. Poult. Sci..

[10] Ta S., Zhao Y., Li H., Stinn S., Xin H., Shepherd T.A., Zhao Y., Li H., Stinn J.P., Hayes M.D., Xin H. (2015). Environmental assessment of three egg production systems—Part II. Ammonia, greenhouse gas, and particulate matter emissions. Poult. Sci..

[11] Thungon P.D., Kakoti A., Ngashangva L., Goswami P. (2017). Advances in developing rapid, reliable and portable detection systems for alcohol. Biosens. Bioelectron..

[12] Timsorn K., Lorjaroenphon Y., Wongchoosuk C. (2017). Identification of adulteration in uncooked Jasmine rice by a portable low-cost artificial olfactory system. Measurement.

[13] Järvinen T., Lorite G.S., Rautio A.-R., Juhász K.L., Kukovecz Á., Kónya Z., Kordas K., Toth G. (2017). Portable cyber-physical system for indoor and outdoor gas sensing. Sens. Actuators B Chem..

[14] Hyodo T., Urata K., Kamada K., Ueda T., Shimizu Y. (2017). Semiconductor-type SnO_2_-based NO_2_ sensors operated at room temperature under UV-light irradiation. Sens. Actuators B Chem..

[15] Wang Z., Peng X., Huang C., Chen X., Dai W., Fu X. (2017). CO gas sensitivity and its oxidation over TiO_2_ modified by PANI under UV irradiation at room temperature. Appl. Catal. B Environ..

[16] Liu Z., Yu L., Guo F., Liu S., Qi L., Shan M., Fan X. (2017). Facial development of high performance room temperature NO_2_ gas sensors based on ZnO nanowalls decorated rGO nanosheets. Appl. Surf. Sci..

[17] Abu-Hani A.F.S., Greish Y.E., Mahmoud S.T., Awwad F., Ayesh A.I. (2017). Low-temperature and fast response H_2_S gas sensor using semiconducting chitosan film. Sens. Actuators B Chem..

[18] Zhuang Z., Qi D., Ru C., Pan J., Zhao C., Na H. (2017). Fast response and highly sensitive humidity sensors based on CaCl_2_-doped sulfonated poly (ether ether ketone)s. Sens. Actuators B Chem..

[19] Piloto C., Mirri F., Bengio E.A., Notarianni M., Gupta B., Shafiei M., Pasquali M., Motta N. (2016). Room temperature gas sensing properties of ultrathin carbon nanotube films by surfactant-free dip coating. Sens. Actuators B Chem..

[20] Andre R.S., Chen J., Kwak D., Correa D.S., Mattoso L.H.C., Lei Y. (2017). A flexible and disposable poly(sodium 4-styrenesulfonate)/polyaniline coated glass microfiber paper for sensitive and selective detection of ammonia at room temperature. Synth. Met..

[21] Su P.-G., Yang L.-Y. (2016). NH_3_ gas sensor based on Pd/SnO_2_/RGO ternary composite operated at room-temperature. Sens. Actuators B Chem..

[22] Wang J., Yang P., Wei X. (2015). High-Performance, Room-Temperature, and No-Humidity-Impact Ammonia Sensor Based on Heterogeneous Nickel Oxide and Zinc Oxide Nanocrystals. ACS Appl. Mater. Interfaces.

[23] Andre R.S., Shimizu F.M., Miyazaki C.M., Riul A., Manzani D., Ribeiro S.J.L., Oliveira O.N., Mattoso L.H.C., Correa D.S. (2017). Hybrid layer-by-layer (LbL) films of polyaniline, graphene oxide and zinc oxide to detect ammonia. Sens. Actuators B Chem..

[24] Shafiei M., Hoshyargar F., Lipton-Duffin J., Piloto C., Motta N., OMullane A.P. (2015). Conversion of n-Type CuTCNQ into p-Type Nitrogen-Doped CuO and the Implication for Room-Temperature Gas Sensing. J. Phys. Chem. C.

[25] Sun P., Cai Y., Du S., Xu X., You L., Ma J., Liu F., Liang X., Sun Y., Lu G. (2013). Hierarchical α-Fe_2_O_3_/SnO_2_ semiconductor composites: Hydrothermal synthesis and gas sensing properties. Sens. Actuators B Chem..

[26] Masson N., Piedrahita R., Hannigan M. (2015). Approach for quantification of metal oxide type semiconductor gas sensors used for ambient air quality monitoring. Sens. Actuators B Chem..

[27] Feng Q., Li X., Wang J., Gaskov A.M. (2016). Reduced graphene oxide (rGO) encapsulated Co_3_O_4_ composite nanofibers for highly selective ammonia sensors. Sens. Actuators B Chem..

[28] Liu X., Cheng S., Liu H., Hu S., Zhang D., Ning H. (2012). A Survey on Gas Sensing Technology. Sensors.

[29] Park S., An S., Ko H., Jin C., Lee C. (2012). Synthesis of nanograined ZnO nanowires and their enhanced gas sensing properties. ACS Appl. Mater. Interfaces.

[30] Deng J., Fu Q., Luo W., Tong X., Xiong J., Hu Y., Zheng Z. (2016). Enhanced H2S gas sensing properties of undoped ZnO nanocrystalline films from QDs by low-temperature processing. Sens. Actuators B Chem..

[31] Qiu Y., Yang M., Fan H., Xu Y., Shao Y., Yang X., Yang S. (2014). Synthesis of ZnO nanorod arrays on Zn substrates by a gas–solution–solid method and their application as an ammonia sensor. J. Mater. Sci..

[32] Ponnusamy D., Madanagurusamy S. (2014). Nanostructured ZnO Films for Room Temperature Ammonia Sensing. J. Electron. Mater..

[33] Li C.-F., Hsu C.-Y., Li Y.-Y. (2014). NH3 sensing properties of ZnO thin films prepared via sol-gel method. J. Alloys Compd..

[34] Guo W., Liu T., Zeng W., Liu D., Chen Y., Wang Z. (2011). Gas-sensing property improvement of ZnO by hierarchical flower-like architectures. Mater. Lett..

[35] Wei A., Pan L., Huang W. (2011). Recent progress in the ZnO nanostructure-based sensors. Mater. Sci. Eng. B.

[36] Arshak K., Moore E., Lyons G.M., Harris J., Clifford S. (2004). A review of gas sensors employed in electronic nose applications. Sens. Rev..

[37] Mercante L.A., Scagion V.P., Migliorini F.L., Mattoso L.H.C., Correa D.S. (2017). Electrospinning-based (bio)sensors for food and agricultural applications: A review. TrAC Trends Anal. Chem..

[38] Pascariu P., Airinei A., Olaru N., Petrila I., Nica V., Sacarescu L., Tudorache F. (2016). Microstructure, electrical and humidity sensor properties of electrospun NiO-SnO_2_ nanofibers. Sens. Actuators B Chem..

[39] Yang X., Salles V., Kaneti Y.V., Liu M., Maillard M., Journet C., Jiang X., Brioude A. (2015). Fabrication of highly sensitive gas sensor based on Au functionalized WO_3_ composite nanofibers by electrospinning. Sens. Actuators B Chem..

[40] Wu H., Pan W., Lin D., Li H. (2012). Electrospinning of ceramic nanofibers: Fabrication, assembly and applications. J. Adv. Ceram..

[41] Piloto C., Shafiei M., Khan H., Gupta B., Tesfamichael T., Motta N. (2018). Sensing performance of reduced graphene oxide-Fe doped WO_3_ hybrids to NO_2_ and humidity at room temperature. Appl. Surf. Sci..

[42] Li W., Wu X., Chen J., Gong Y., Han N., Chen Y. (2017). Abnormal n-p-n type conductivity transition of hollow ZnO/ZnFe_2_O_4_ nanostructures during gas sensing process: The role of ZnO-ZnFe_2_O_4_ hetero-interface. Sens. Actuators B Chem..

[43] Song X.-Z., Qiao L., Sun K.-M., Tan Z., Ma W., Kang X.-L., Sun F.-F., Huang T., Wang X.-F. (2018). Triple-shelled ZnO/ZnFe_2_O_4_ heterojunctional hollow microspheres derived from Prussian Blue analogue as high-performance acetone sensors. Sens. Actuators B Chem..

[44] Li Y., Luo N., Sun G., Zhang B., Ma G., Jin H., Wang Y., Cao J., Zhang Z. (2018). Facile synthesis of ZnO/ZnFe_2_O_4_/α-Fe_2_O_3_ porous microrods with enhanced TEA-sensing performance. J. Alloys Compd..

[45] Poloju M., Jayababu N., Ramana Reddy M.V. (2018). Improved gas sensing performance of Al doped ZnO/CuO nanocomposite based ammonia gas sensor. Mater. Sci. Eng. B.

[46] Pang Z., Yang Z., Chen Y., Zhang J., Wang Q., Huang F., Wei Q. (2016). A room temperature ammonia gas sensor based on cellulose/TiO_2_/PANI composite nanofibers. Colloids Surf. A Physicochem. Eng. Asp..

[47] Lin Y., Huang L., Chen L., Zhang J., Shen L., Chen Q., Shi W. (2015). Fully gravure-printed NO_2_ gas sensor on a polyimide foil using WO_3_-PEDOT:PSS nanocomposites and Ag electrodes. Sens. Actuators B Chem..

[48] Liu Y., Ding Y., Zhang L., Gao P.-X., Lei Y. (2012). CeO_2_ nanofibers for in situ O_2_ and CO sensing in harsh environments. RSC Adv..

[49] Liu Y., Sun X., Zhou Z., Lei Y. (2014). Electrospun Ce-Ni-O composite nanofibers for highly selective propane detection at high temperature based on its rapid reaction kinetics. J. Mater. Chem. A.

[50] Wang Y., Ding Y., La A., Lei Y., Mason E.C., Weber A.P. (2011). Preparation, characterization and gas sensing application of novel conducting polypyrrole composite nanofibers. Polypyrrole: Properties, Performance and Applications.

[51] Rahman M.M., Khan S.B., Marwani H.M., Asiri A.M., Alamry K.A., Rub M.A., Khan A., Khan A.A.P., Azum N. (2014). Facile synthesis of doped ZnO-CdO nanoblocks as solid-phase adsorbent and efficient solar photo-catalyst applications. J. Ind. Eng. Chem..

[52] Cai X., Hu D., Deng S., Han B., Wang Y., Wang Y., Wu J. (2014). Isopropanol sensing properties of coral-like ZnO-CdO composites by flash preparation via self-sustained decomposition of metal-organic complexes. Sens. Actuators B Chem..

[53] Park S.Y., Kim S., Yoo J., Lim K.-H., Lee E., Kim K., Kim J., Kim Y.S. (2014). Aqueous zinc ammine complex for solution-processed ZnO semiconductors in thin film transistors. RSC Adv..

[54] Hsieh P.-T., Chen Y.-C., Kao K.-S., Wang C.-M. (2007). Luminescence mechanism of ZnO thin film investigated by XPS measurement. Appl. Phys. A.

[55] Seekaew Y., Lokavee S., Phokharatkul D., Wisitsoraat A., Kerdcharoen T., Wongchoosuk C. (2014). Low-cost and flexible printed graphene-PEDOT:PSS gas sensor for ammonia detection. Org. Electron..

[56] Xu S., Kan K., Yang Y., Jiang C., Gao J., Jing L., Shen P., Li L., Shi K. (2015). Enhanced NH_3_ gas sensing performance based on electrospun alkaline-earth metals composited SnO_2_ nanofibers. J. Alloys Compd..

[57] Bai S., Tian Y., Cui M., Sun J., Tian Y., Luo R., Chen A., Li D. (2016). Polyaniline@SnO_2_ heterojunction loading on flexible PET thin film for detection of NH_3_ at room temperature. Sens. Actuators B Chem..

[58] Sundara Venkatesh P., Dharmaraj P., Purushothaman V., Ramakrishnan V., Jeganathan K. (2015). Point defects assisted NH_3_ gas sensing properties in ZnO nanostructures. Sens. Actuators B Chem..

[59] Lin Q., Li Y., Yang M. (2012). Tin oxide/graphene composite fabricated via a hydrothermal method for gas sensors working at room temperature. Sens. Actuators B Chem..

[60] Loyon L., Burton C.H., Misselbrook T., Webb J., Philippe F.X., Aguilar M., Doreau M., Hassouna M., Veldkamp T., Dourmad J.Y. (2016). Best available technology for European livestock farms: Availability, effectiveness and uptake. J. Environ. Manag..

[61] Lou Z., Deng J., Wang L., Wang R., Fei T., Zhang T. (2013). A class of hierarchical nanostructures: ZnO surface-functionalized TiO_2_ with enhanced sensing properties. RSC Adv..

[62] Wang T., Kou X., Zhao L., Sun P., Liu C., Wang Y., Shimanoe K., Yamazoe N., Lu G. (2017). Flower-like ZnO hollow microspheres loaded with CdO nanoparticles as high performance sensing material for gas sensors. Sens. Actuators B Chem..

[63] Lim Y.T., Son J.Y., Rhee J.-S. (2013). Vertical ZnO nanorod array as an effective hydrogen gas sensor. Ceram. Int..

[64] Rai P., Khan R., Ahmad R., Hahn Y.-B., Lee I.-H., Yu Y.-T. (2013). Gas sensing properties of single crystalline ZnO nanowires grown by thermal evaporation technique. Curr. Appl. Phys..

[65] Gurav K.V., Patil U.M., Shin S.W., Pawar S.M., Kim J.H., Lokhande C.D. (2012). Morphology evolution of ZnO thin films from aqueous solutions and their application to liquefied petroleum gas (LPG) sensor. J. Alloys Compd..

[66] Navaneethan M., Patil V.L., Ponnusamy S., Muthamizhchelvan C., Kawasaki S., Patil P.S., Hayakawa Y. (2018). Sensitivity enhancement of ammonia gas sensor based on Ag/ZnO flower and nanoellipsoids at low temperature. Sens. Actuators B Chem..

[67] Patil N.B., Nimbalkar A.R., Patil M.G. (2018). ZnO thin film prepared by a sol-gel spin coating technique for NO_2_ detection. Mater. Sci. Eng. B.

[68] Wang Y., Akhigbe J., Ding Y., Brückner C., Lei Y. (2012). meso-Tritolylcorrole-Functionalized Single-walled Carbon Nanotube Donor Acceptor Nanocomposites for NO_2_ Detection. Electroanalysis.

[69] Ganbavle V.V., Inamdar S.I., Agawane G.L., Kim J.H., Rajpure K.Y. (2016). Synthesis of fast response, highly sensitive and selective Ni:ZnO based NO_2_ sensor. Chem. Eng. J..

[70] Wang Y., Jia W., Strout T., Ding Y., Lei Y. (2009). Preparation, Characterization and Sensitive Gas Sensing of Conductive Core-sheath TiO_2_-PEDOT Nanocables. Sensors.

